# Removal of obstructive organized tracheo-bronchial clots under respiratory support using veno-venous extracorporeal membrane oxygenation in a patient with blunt thoracic trauma: a case report

**DOI:** 10.1186/s44215-023-00080-z

**Published:** 2023-08-01

**Authors:** Atsushi Tanikawa, Motoo Fujita, Yasushi Kudo, Ken Katsuta, Yoshiaki Kurokawa, Takeaki Sato, Shigeki Kushimoto

**Affiliations:** 1 Department of Emergency and Critical Care Medicine, Tohoku University Hospital, 1-1 Seiryo-Machi, Aoba-Ku, Sendai, 980-8574 Japan; 2 Division of Emergency and Critical Care Medicine, Tohoku University Graduate School of Medicine, 1-1 Seiryo-Machi, Aoba-Ku, Sendai, 980-8574 Japan

**Keywords:** Thoracic trauma, Endotracheal blood clot, Extracorporeal membrane oxygenation

## Abstract

**Background:**

It has been reported that veno-venous extracorporeal membrane oxygenation is useful for treating patients with acute respiratory failure following severe thoracic trauma. However, the removal of an obstructive organized tracheo-bronchial clot under respiratory support using extracorporeal membrane oxygenation in a patient with trauma has never been reported.

**Case presentation:**

A teenage female was injured in a fall and experienced right open hemopneumothorax and bilateral lung contusions. Since she was in refractory shock due to bleeding into the right thoracic cavity, we performed right thoracotomy and resection of the lacerated right middle lobe. After hemorrhage control, her respiratory status could not be maintained under mechanical respiratory support. Therefore, we initiated veno-venous extracorporeal membrane oxygenation. Although her respiratory failure gradually improved, the removal of obstructing tracheo-bronchial organized blood clots using bronchoscopy was required daily. Because of the size and firm adherence of organized clots to the airway membrane, we performed a tracheostomy to remove organized clots directly through the incision under extracorporeal membrane oxygenation without any adverse events on day 9. The next day, she was successfully removed from extracorporeal membrane oxygenation.

**Conclusions:**

Extracorporeal membrane oxygenation could provide respiratory support not only for acute respiratory failure but also for removal of obstructing tracheo-bronchial organized clots in patients with severe lung injury following trauma.

## Background

Organized blood clots caused by hemorrhage in tracheo-bronchus is life threatening [1, 2]. To avoid fatal sequelae from airway compromise, hemorrhage control and clot removal are mandatory [3, 4]. For patients with acute respiratory failure due to severe chest trauma, veno-venous extracorporeal membrane oxygenation (VV-ECMO) could be an option to maintain respiratory status [5–9]. Although there are several reports regarding the usefulness of VV-ECMO during the acute phase of management of patients with acute respiratory failure following chest trauma [5–9], to our knowledge, no reports have been found on the use of VV-ECMO for the removal of obstructive organized tracheo-bronchial clots from patients with trauma under respiratory support.

Therefore, we present a case of a patient with obstructive organized bronchial clots due to chest trauma, for whom clot removal was successfully performed under respiratory support using VV-ECMO without any complications.

## Case presentation

**Fig. 1:**
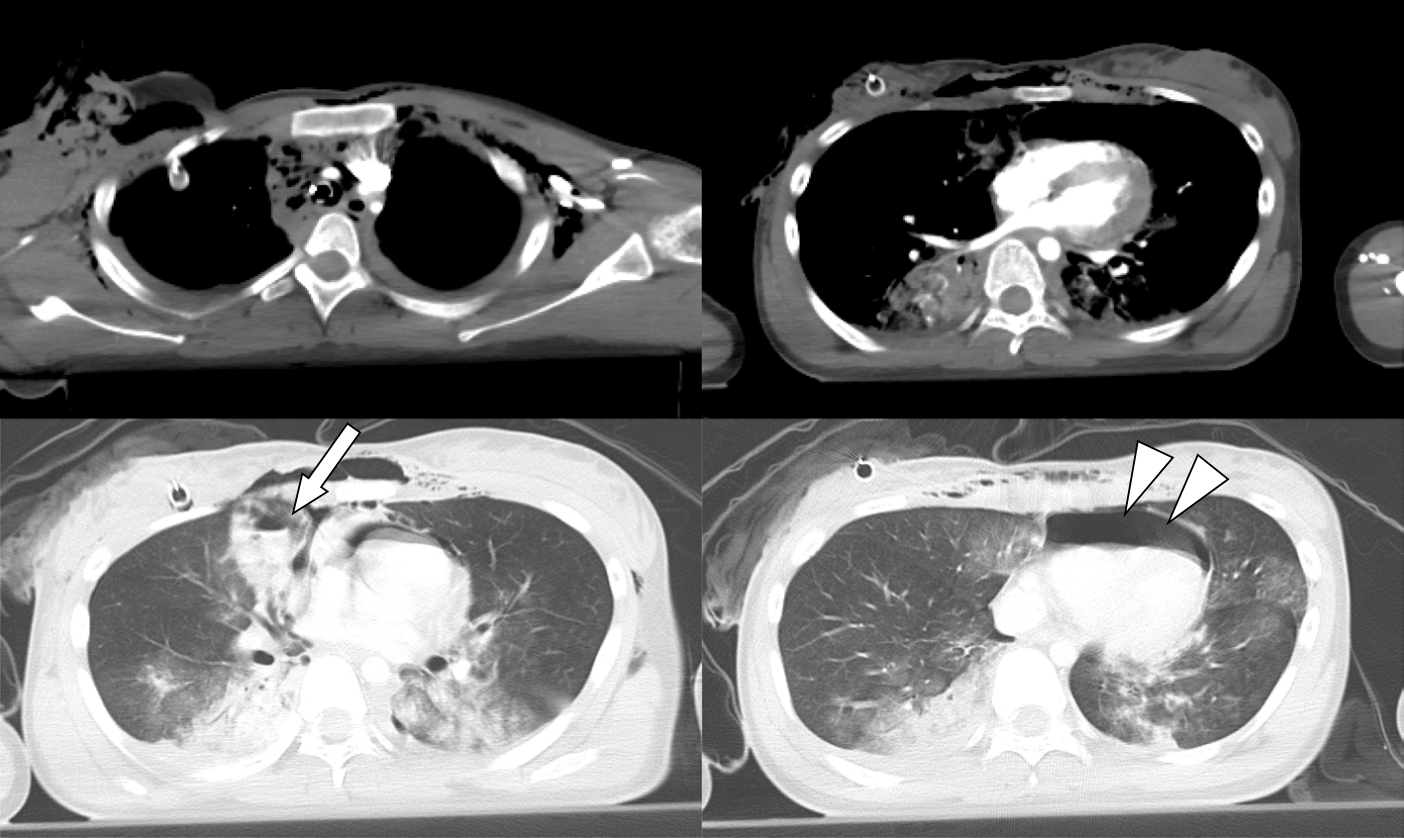
Computed tomography findings on admission. Computed tomography shows an intraparenchymal laceration of the right middle lobe (arrow) and pericardial emphysema (arrowheads)

A teenage female was injured in a fall from a height of approximately 30 m and was transported to our emergency department by helicopter medical service. Upon arrival at our institution, approximately 1 h after injury, her Glasgow coma score was 3 after drug-assisted intubation during prehospital care, blood pressure was 68/26 mmHg, heart rate was 157 bpm, and respiratory rate was 40 breaths/min. A chest tube had been inserted for a right open hemopneumothorax, presenting with a 1500 mL of hemorrhage from the drainage tube at the time of arrival. We have a novel trauma resuscitation room, named “Hybrid Emergency Room” that is equipped with a whole-body computed tomography (CT) scanner, and an angiography system. It enables innovative trauma workflow, in which whole-body CT, emergency surgery, and endovascular treatment are immediately and sequentially conducted without the necessity for time-consuming patient transfer. We performed whole-body CT while we resuscitated her and prepared for surgery. The CT showed cerebral contusion, extensive laceration and intraparenchymal hemorrhage of the right middle lobe, bilateral extensive lung contusions, right open hemopneumothorax, pneumopericardium, and multiple rib fractures (Fig. 1). We performed right thoracotomy to control thoracic hemorrhage and release pneumopericardium. Due to the extensive laceration and intraparenchymal hemorrhage of the right middle lobe, preservation of the damaged lobe was considered unsuitable. Therefore, the right middle lobe was resected with a linear cutter stapler (ETHICON™, USA), and laceration and air leakage of the bronchus of the right upper lobe was repaired using the lateral suture technique. No significant cardio-pericardial injury was observed at the time of pericardiotomy, and pneumopericardium was assessed to be caused by a combination of minor pericardial tear and pneumothorax. Hemorrhage and air leakage in the right thorax were controlled; however, the blood gas analysis revealed a pH of 7.01, a PO_2_ 62.3 mmHg, and a PCO_2_ of 89.1 mmHg under positive pressure mechanical ventilation with an F_i_O_2_ of 1.0, and positive end-expiratory pressure (PEEP) of 20 cmH_2_O. Therefore, VV-ECMO (MERA centrifugal blood pump system HAS-CFP; SENKO Medical Instrument, Tokyo, Japan; venous return cannula (16 Fr.) in the right internal jugular vein, outflow cannula (21 Fr.) in the right femoral vein using heparin-bonded catheters) was applied to maintain respiratory status without systemic heparinization in the hybrid emergency room before admitting the patient to the intensive care unit.

**Fig. 2:**
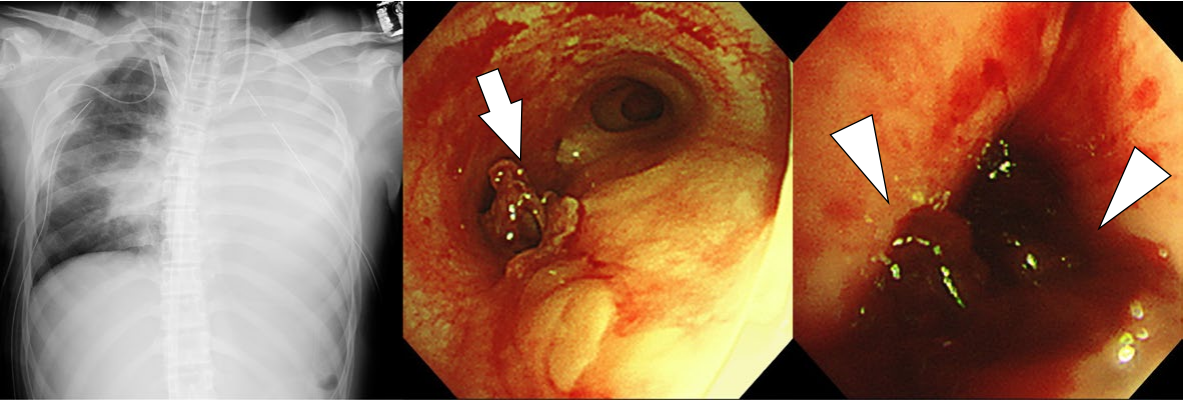
X-ray and bronchoscopy findings on the 6th day. The radiograph shows whole atelectasis of the left lung, and bronchoscopy shows the left bronchus obstructed by an organized bronchial clot (arrow) and an old hematoma in the left peripheral bronchus (arrowheads)

**Fig. 3:**
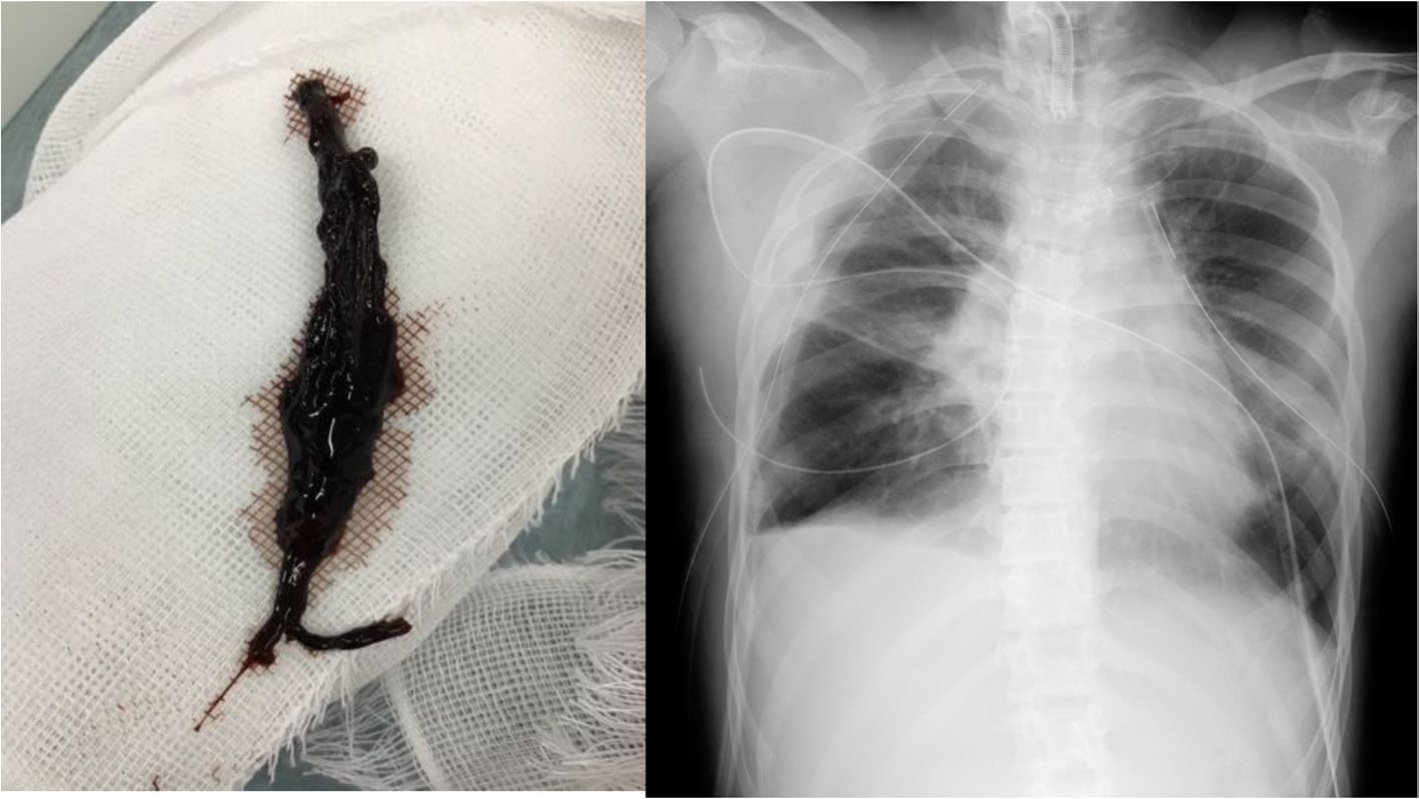
Organized blood clot removal from tracheostomy site and radiograph after veno-venous extracorporeal membrane oxygenation withdrawal. The organized large clot was removed on day 9 (left), and apparent atelectatic was not observed in the left lung field after weaning from veno-venous extracorporeal membrane oxygenation on day 10 (right)

She required daily removal of endotracheal organized blood clots due to extensive pulmonary and bronchus contusions using bronchoscopy for consecutive days, to restore airway ventilation. We started systemic heparinization with a target activated partial thromboplastin time between 50 and 80 s from day 4. As the patient’s left pulmonary contusion was relatively minor, we did not consider surgical intervention for hemostasis. However, the left main bronchus became occluded with a hematoma and required daily clot removal especially after systemic heparinization was initiated (Fig. 2). Because of the size and firm adherence of organized clots to the airway membrane and contact bleeding during systemic heparinization, we found it difficult to remove the organized clots via bronchoscopy under oral intubation. Based on these clinical findings, we tried to remove the organized tracheo-bronchial clots directly through a tracheostomy incision on day 9. We first performed typical tracheostomy through a 3-cm-long transverse incision with inverse U-shaped tracheal opening. Then, we located the organized clots in the left main bronchus via bronchoscopy under oral intubation and directly removed the clots through the incision using DeBakey forceps blindly. We completed the tracheostomy and removal of occlusive organized clots under ECMO (blood flow 3.1 L/min, sweep gas flow 4 L/min, rotational speed 3100 rpm, and F_i_O_2_ 0.9) without any complications in a procedure of approximately 100 min during which her respiratory status was maintained without aggressive ventilator support, using pressure control ventilation setting with PIP 15 cmH_2_O, PEEP 10 cmH_2_O, RR 10/min, and F_i_O_2_ 0.4. Thereafter, her lung ventilation was restored immediately, and the radiograph finding was improved (Fig. 3). The next day, she was successfully weaned from ECMO.

On day 22, she was withdrawn from the ventilator, and on day 90, the tracheostomy tube was removed. Finally, she was transferred to the rehabilitation hospital with no residual neurological deficits on day 102.

## Discussion

We described a patient with blunt trauma who underwent removal of organized tracheo-bronchial clots safely without any complications while receiving respiratory support with VV-ECMO following severe chest trauma.

The use of VV-ECMO for patients with acute respiratory failure following trauma has been frequently reported [5–9]. However, a systematic review of ECMO utilization in trauma injuries reported that the mortality rate of using ECMO in patients with blunt trauma was approximately 30% [10]. The ELSO guidelines suggest that adult VV-ECMO for thoracic trauma should be indicated in specific clinical conditions such as traumatic lung injury and severe pulmonary contusion [11].

Airway obstruction caused by massive bleeding or blood clots is life threatening [1, 2]. The respiratory status of our patient could not be maintained with only mechanical ventilation and we had difficulty in removing organized tracheo-bronchial clots. Owing to secured oxygenation under ECMO, we repeatedly tried to remove the blood clots by bronchoscopy and finally succeeded in removing the clots under tracheostomy without any adverse events. Although some reports showed that bronchoscopic cryotherapy may be effective for the removal of tracheo-bronchial clots [2, 12], removing clots safely under definite oxygenation status with ECMO might be an option for critically ill patients with airway obstructions for whom clot removal could potentially be life threatening.

The usefulness of ECMO in the treatment of acute respiratory failure following traumatic airway hemorrhage is also dependent on the subsequent procedures used immediately to control bleeding and relieve airway obstruction. It would be important to perform rapid control of bleeding source and relieve airway obstruction. In the present case, although the major source of airway bleeding was surgically controlled at the time of the initial operation, minor hemorrhage and clot formation caused by extensive bilateral pulmonary contusion may have continued thereafter, which, along with clot lysis, may have contributed to recurrent airway compromise. As seen in this case, ECMO may help in gaining time to improve respiratory status and airway problems.

ECMO may be an effective modality for patients with a higher risk of airway obstruction as well as acute respiratory failure. Although the efficacy of ECMO prior to intubation and general anesthesia for patients with a higher risk of respiratory decompensation has been reported [13], the usefulness of ECMO for removing tracheo-bronchial clots as well as support for respiratory failure in patients with trauma as in this case has never been reported. Further case accumulation and assessment would be required to validate the findings.

In conclusion, ECMO may provide respiratory support not only for acute respiratory failure but also for removal of obstructing tracheo-bronchial organized clots in patients with severe chest injury following trauma.

## Data Availability

Data sharing is not applicable to this article, as no datasets were generated or analyzed during the current study.

## Abbreviations

VV-ECMO: Veno-venous extracorporeal membrane oxygenation
PCO_2_: Partial pressure of arterial carbon dioxide
PO_2_: Partial pressure of arterial oxygen
F_i_O_2_: Fraction of inspiratory oxygen
PEEP: Positive end-expiratory and pressure
PIP: Peak inspiratory pressure
RR: Respiratory rate
